# The p53 Isoforms as Potential Biomarkers in Different Cancer Entities

**DOI:** 10.3390/ijms27125153

**Published:** 2026-06-06

**Authors:** Christine Supina Pavić, Anđela Horvat, Ana Tadijan, Janja Josić, Martina Deželjin, Maja Jazvinšćak Jembrek, Ignacija Vlašić, Neda Slade

**Affiliations:** 1 Laboratory for Protein Dynamics, Division of Molecular Medicine, Ruđer Bošković Institute, Bijenička cesta 54, 10000 Zagreb, Croatia; christine.supina@irb.hr (C.S.P.); andjela.horvat@irb.hr (A.H.); janja.josic@irb.hr (J.J.); maja.jazvinscak.jembrek@irb.hr (M.J.J.); 2 Laboratory for Molecular and Cellular Biology, Division of Molecular Biology, Ruđer Bošković Institute, Bijenička cesta 54, 10000 Zagreb, Croatia; ana.tadijan@irb.hr (A.T.); martina.dezeljin@irb.hr (M.D.); 3 School of Medicine, Catholic University of Croatia, Ilica 244, 10000 Zagreb, Croatia

**Keywords:** p53 isoforms, tumor suppressor p53, cancer

## Abstract

The p53 protein is a pivotal tumor suppressor that is mutated in more than half of tumor cases in humans. In addition, its activity/function can be perturbed by various other mechanisms. Existence of two promoters in the *TP53* gene and extensive splicing on N- and C-terminus, as well as alternative translation initiation, give rise to numerous p53 protein isoforms. Different p53 protein isoforms can form heterotetramers with canonical full-length p53 or compete in binding target genes’ promoters as tetramers which can result in modulation of p53 function. In this review we have gathered the most novel research on the p53 isoform network including the isoforms’ expression profiles and biological functions in the most frequent cancer types. The expression of p53 isoforms differs among tumor types and compared with normal tissues, thereby affecting biological processes associated with tumorigenesis, such as apoptosis, cell cycle regulation, migration, senescence, stemness, etc. We also discussed the potential of targeting p53 isoforms by direct mechanisms that can change the ratio between specific isoforms and thus modulate their activity or indirectly by targeting downstream pathways regulated by a specific isoform. More profound understanding of the p53 pathway regulation could contribute to improvement in current therapies.

## 1. Introduction

Considered as the guardian of the genome, p53 protein plays a central role in maintaining genome stability [1]. As a tumor suppressor, p53 has an important function in restraining tumorigenesis by regulating cellular processes and different cell fate decisions in response to oncogenic stimuli. Depending on the stress signals in diverse cell types, p53 can activate DNA-repair machinery and promote cell survival, induce senescence or apoptosis, and/or coordinate different signaling pathways. Therefore, functioning as transcription factor, p53 concurrently gathers abundant cell signals and assists in their rendering into cellular responses specific to the type and context of the cell [2,3]. Although p53 functions as a tumor suppressor, some of its biochemical functions can display oncogenic effects. In cancer cells, the inactivation of tumor suppressors usually occurs via nonsense mutations or gene deletions. However, *TP53* frequently undergoes missense mutations that often lead in gain-of-function (GOF) effects which can influence carcinogenesis [4,5]. The scope of the cellular functions mediated by p53 is more complex due to the presence of p53 isoforms.

**Figure 1:**
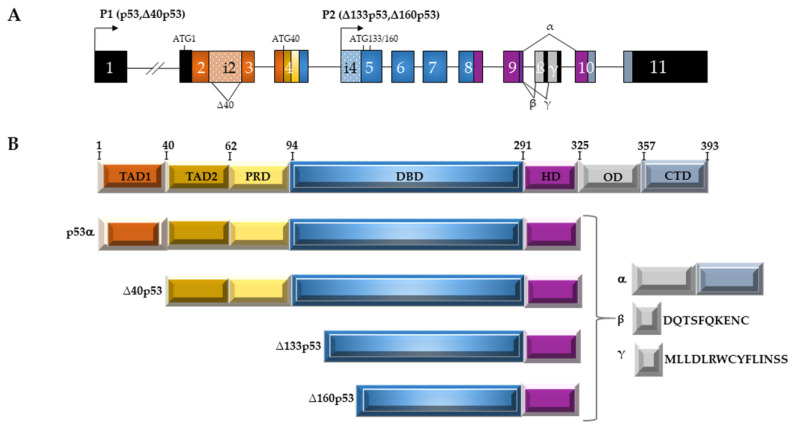
*TP53* gene structure (**A**) and functional domains of p53 protein and its isoforms (**B**) (for details, see the text).

Transcription from alternative promoters, alternative splicing and translation from diverse initiation sites generate co-expressed p53 isoforms that differ in N- and/or C-terminus and thus have different activities (Figure 1). Their activities partly arise from the ability to form tetramers and govern transcription of specific target genes. Due to their capacity to interact, the isoforms can inflect the functions of canonical, full-length p53 (FLp53) protein [6,7]. While some p53 isoforms stimulate the function of FLp53, others have the ability to impair it. Although individual p53 isoforms are not strictly considered as either oncogenes or tumor suppressors, their imbalance can result in pathological conditions, such as cancer. Even though the expression of p53 isoforms is tissue-specific, it is often dysregulated in various cancers [8].

Therefore, imbalance between p53 isoforms is shown to be associated with cancer cell development, progression and resistance to therapy which makes them promising targets for cancer treatment [6,9,10,11,12]. This review summarizes biological functions of p53 isoforms in different cancer entities, highlighting novel findings and possibilities of their targeting, with a focus on strategies to improve current therapeutic approaches.

## 2. p53 Isoforms and Their Roles in Different Cancer Entities

The *TP53* gene consists of 11 exons, two alternative exons (9β and 9γ) and 10 introns (Figure 1A). It possesses a dual-gene structure, having two distinct but joined transcriptional units. Canonical promoter P1 is situated upstream of exon 1, whereas alternative promoter P2 is located in intron 4. In addition to usage of alternative promoters, alternative splicing at N- and C-termini and alternative translation initiation sites give rise to multiple p53 mRNA isoforms. In consequence, aside from FLp53 protein, which is also referred to as canonical p53, TAp53 or p53α, specific p53 protein isoforms differ in N- and/or C-termini and thus are impaired in some of the functional domains. The full-length p53α isoform contains seven functional domains which include two transactivation domains (TAD1 and TAD2), a proline-rich domain (PRD), a DNA-binding domain (DBD), a hinge domain (HD), an oligomerization domain (OD) and a C-terminal domain (CTD) (Figure 1B). Depending on whether first 39, 132 or 159 amino acids are missing at the N-terminus, the isoforms are designated as Δ40p53, Δ133p53 or Δ160p53, respectively. Isoforms Δ40p53 lack TAD1, while Δ133p53 and Δ160p53 are missing both TAD1 and TAD2, PRD and part of the conserved cysteine box of the DBD. Depending on the differences at the C-terminus, the isoforms are classified as α, β or γ. Contrary to α isoforms which contain all functional domains on C-terminus, β and γ isoforms are C-terminally truncated isoforms and lack part of the OD as well as the entire CTD. Accordingly, twelve p53 isoforms differ in the composition of the structural and functional domains and therefore can exhibit diverse functional features (for details, see the following reviews [6,7,10]).

Functional domains are responsible for specific biological functions. For example, TADs are transactivation domains with the ability to activate transcription of target genes [13]. Together, TAD2 and PRD can modulate apoptosis, while PRD alone is necessary for apoptosis activation [14,15,16]. The DBD contains four conserved regions crucial for binding to DNA and for regulating p53 conformation stability. Therefore, the absence of DBD can impair protein structure and provoke aggregation capacity [17]. In addition, frequent missense mutations in DBD, also known as “hotspot“ mutations, are able to hinder binding ability of p53 to DNA thereby impairing expression of p53-target genes [18]. The OD is responsible for p53 tetramerization and subcellular localization dependent on nuclear export signal (NES) [19]. The OD also regulates DNA binding, protein stability and cellular outcome as many OD missense mutants are predicted to disrupt the ability of p53 protein to form tetramers [20]. Although the deficiency of tetramerization propensity is associated with the loss of transcriptional activity, there are some exceptions. Recent findings have shown that some OD mutants are unable to form tetramers but shape stable dimers that display neomorphic activities and altered transcriptional and metabolic profile, as well as both gain-of- and loss-of-function properties [21,22]. The last domain, CTD, contains sites that can be post-translationally modified by various mechanisms, e.g., acetylation, ubiquitination, neddylation, methylation and sumoylation, and therefore regulates the stability and activity of p53 [23]. The loss of functional domains in truncated p53 isoforms alters their structure relative to canonical p53 as well as function which can potentially switch from tumor-suppressive to even oncogenic. Therefore, due to structural destabilization, both Δ133p53 and Δ160p53 have the propensity to form aggregates over heterotetramers with FLp53 thus disrupting its structure and hindering function, ultimately exhibiting a dominant-negative effect [24]. Not surprisingly, N-terminally truncated p53 isoforms have been shown to contribute to cancer development and progression [25].

**Figure 2:**
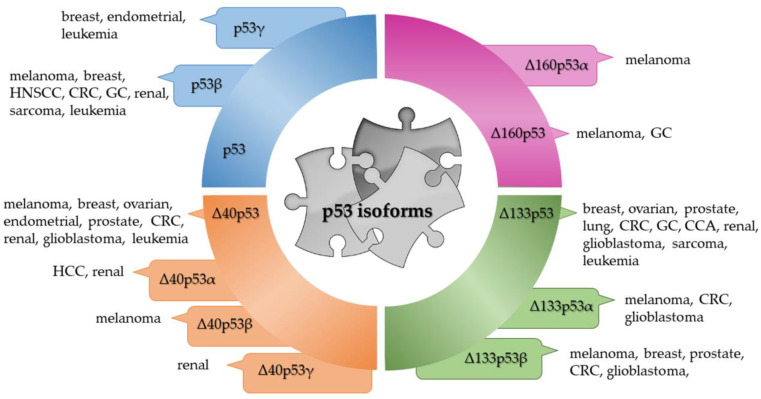
Overview of p53 isoforms whose status (expression or/and functional) has been investigated in particular cancer entities. Abbreviations: HNSCC (head and neck squamous cell carcinoma), CRC (colorectal cancer), GC (gastric cancer), CCA (cholangiocarcinoma).

Some of the biological functions related to cancer development and progression which are associated with N-terminally truncated p53 isoforms, such as Δ40p53 or Δ133p53, include promotion of cell cycle progression, migration and invasion [26,27], angiogenesis [28], DNA repair [29], stemness and chemoresistance [30,31,32], and telomerase activity [33] as well as inhibition of senescence and cell death [29,34]. One of the most influential studies which provided the solid evidence that p53 isoforms are related to the inflammatory process was conducted on mouse models that solely express the N-terminally truncated Δ133p53 isoform [27,35,36]. The Δ122p53 mice, a model of human Δ133p53, have shown to be highly tumor-prone, mostly developing tumors of B-cell origin or bone cancers, and exhibit proinflammatory phenotypes, e.g., lymphocyte aggregation in different organs, extramedullary hematopoiesis, prominent Peyer patches in the colon, and generalized inflammation. Furthermore, increased proinflammatory serum cytokines, especially IL-6 but also IL-3, IL-5 interferon-γ, tumor necrosis factor-α, and several chemokines, were observed in Δ122p53 compared to p53^+/+^ and p53^−/−^ mice [35]. An additional study using the Δ122p53 mouse model showed that proinflammatory cytokine IL-6 and chemokines CCL2 contribute to migratory potential of Δ122p53 expressing cancer cells [27], which could suggest that Δ133p53 can contribute to cancer by promoting inflammation [36]. As expected, many studies have shown that p53 isoforms have differential expression in cancer cells and tissues compared to healthy cells and tissues, and are involved in cancer progression or therapy resistance (Figure 2).

For example, elevated expression of Δ133p53 has been reported in many cancers, such as melanoma [37], breast cancer [26,38], colorectal cancer [26,34,39,40], gastric cancer [41], lung cancer [42] and others, often with more aggressive features and worse clinical outcome [26,37,39,40,43]. Therefore, p53 isoforms may contain the biomarker capacity either of predictive or prognostic relevance and thus have the potential to be targeted. The expression profiles of p53 isoforms and their biological roles in the context of specific cancer types will be discussed in the following sections.

### 2.1. p53 Isoforms in Colorectal Carcinoma

Colorectal cancer (CRC) is the third-most-common type of cancer globally and the second leading cause of cancer-related deaths [44]. The *TP53* gene is frequently mutated in various human tumors and CRC is among tumor types with a high prevalence of *TP53* mutations, with approximately 43% of CRCs harboring p53 mutations according to the *TP53* database (https://tp53.cancer.gov/, accessed on 29 May 2026).

The p53 family isoforms are expressed in both normal and colorectal carcinoma tissue. Their dysregulated expression in tumor cells leads to altered functional activity of p53, influencing tumor formation, progression, and therapeutic response. Moreover, the diverse expression patterns of N- and C-terminal splice variants in healthy tissue, premalignant lesions, and tumors indicate an important role in cancer progression. In normal colon tissue, p53β and γ are expressed at low levels, Δ133p53γ at moderate level, and p53α, as well as Δ133p53α and β isoforms, at high levels [38]. Premalignant lesions of colon adenomas have been shown to express reduced levels of Δ133p53α and elevated levels of p53β compared with normal colon tissue [34,45]. An interesting expression dynamic has been observed during the progression of colon carcinoma; the expression of Δ133p53 increases from stage I to II, whereas that of p53β decreases from stage II to III [34]. Notably, these differences appear to influence progression from colorectal adenoma to carcinoma and regulate cellular senescence [34,45,46,47,48,49,50]. A specific isoform expression pattern in colon adenoma, characterized by high levels of p53β and low levels of Δ133p53, is considered to be senescence-associated. This expression pattern is lost in colorectal carcinoma and may represent a mechanism by which tumor cells evade senescence, leading to carcinoma development [34]. In addition, colon carcinoma tissues can co-express both p73 and p53 proteins, which interact and cooperate to regulate the expression of mutual target genes. For example, in HCT116 cells, p53 and TAp73 cooperatively regulate the expression of PUMA and p21 by simultaneously binding to their promoters, while in p53-depleted HCT116 colon cancer cells, Δ133p53 and TAp73 synergistically promote the expression of DNA-repair genes [51,52].

Recently published studies have shown that different isoforms are involved in various tumorigenic processes, such as autophagy, apoptosis, and DNA repair. Specifically, it has been demonstrated that the Δ40p53 isoform negatively regulates the expression of the transcriptional repressor protein YY1 and inhibits proliferation of p53-null HCT116 cells, while 3′–5′ exonuclease activity of Δ40p53 inhibits autophagy [53,54]. Additionally, Δ133p53β isoform inhibits camptothecin-induced apoptosis in CRC cells by binding to a tumor suppressor RhoB (Ras homolog family member B) [39]. It was shown that Δ133p53 and TAp73α accumulate in HCT116 cells following γ-irradiation and promote the expression of RAD51, LIG4, and RAD52 leading to DNA double-strand break repair [52]. Furthermore, Δ133p53 regulates the CRC invasion. Invasive CRC cell lines express elevated levels of Δ133p53 isoforms, and specifically Δ133p53α, Δ133p53β, and Δ133p53γ promote the invasion of HCT116 cells through matrigel via RhoA -ROCK (Rho-associated protein kinase A) pathway [40]. Moreover, HCT116 cells expressing only Δ133p53 isoforms are more invasive, whereas depletion of p53β reduces invasiveness in vitro. HCT116 cells ectopically expressing Δ133p53 isoforms show increased expression of interleukins 6 and 8, as well as the anti-apoptotic protein Bcl-2 [55]. In addition to activating RhoA-ROCK pathway, interleukin 6 also activates the JAK-STAT3 pathway, both of which promote tumor cell invasion and metastasis. Therefore, it is not surprising that increased expression of Δ133p53 mRNA is associated with distant recurrence and poor patient survival [40]. Furthermore, HCT116 cells ectopically expressing the Δ133p53β isoform initiate epithelial–amoeboid transition, leading to a more invasive phenotype [26]. Higher levels of Δ133p53β in CRC are associated with more invasive tumors, shorter disease-free survival (DFS), increased recurrence, poor patient outcome, and altered immune cell infiltration [40]. As already mentioned, not only the expression levels of individual p53 isoforms but also the ratio of different p53 isoforms have significant prognostic value. For example, CRC patients with a high Δ133p53/p53α mRNA ratio have a more advanced disease [34]. Based on these results, it appears that the ratio between different p53 isoforms, including Δ133p53, determines the invasive potential of CRC and that specifically levels of Δ133p53β could predict patients’ response to immunotherapy as well as identify patients with aggressive disease.

Germline and somatic p53 family variants play a crucial role in tumorigenesis. Notably, a germline variant affecting p53β isoforms predisposes individuals to familial CRC. In a whole-exome sequencing cohort of 94 individuals suspected of having a genetic predisposition to CRC, a heterozygous germline stop-lost variant was identified in the cryptic exon 9β of the p53 gene, affecting the p53β isoforms. This stop-lost variant results in a 17 amino-acid extension of the p53β isoforms, which increases oligomerization with canonical p53α and dysregulates the expression of p53 target genes. This study highlights the ability of p53β mutants to influence p53 signaling and contribute to susceptibility to various cancer types, including CRC [56].

Early detection of cancer is essential for achieving an effective response to therapy and consequently reducing cancer-related mortality. More than 63% of CRC cases are diagnosed at late stages (III and IV) when surgery is insufficient, tumor cells have metastasized, and patient survival rates are very low. In recent years, circulating biomarkers associated with CRC, such as circulating tumor cells (CTC), circulating tumor DNA (ctDNA), proteins, and autoantibodies, have attracted considerable interest, as they can be detected by liquid biopsy from the blood and used as diagnostic or prognostic biomarkers, as well as markers of response to treatment [57,58]. The humoral immune response associated with cancer is recognized as an attractive approach for early cancer diagnosis [59,60,61]. Specifically, autoantibodies produced against specific tumor-associated autoantigens can be detected in plasma or serum samples and offer several advantages for diagnosis. The p53 protein family is known to be involved in several tumorigenic processes, such as cell differentiation, proliferation, apoptosis, tumor progression, and metastasis [62]. However, the role of the various p53 family isoforms in cancer initiation, progression, and metastasis has not been elucidated [62,63,64]. As the protein expression levels of p53 isoforms are tightly regulated during cancer formation and progression, they can trigger a specific humoral immune response, which may be useful for blood-based diagnosis of CRC. A recently published study, in which seroreactivity assays were performed, confirmed that most seroreactive p53 isoforms can be used to differentiate CRC patients and individuals with premalignant lesions from healthy individuals. Among the p53 isoforms, p53γ, Δ40p53β, Δ40p53γ, Δ133p53γ, and Δ160p53γ showed higher differential seroreactivity in CRC patients and individuals with colorectal premalignant lesions compared with healthy individuals. Specifically, autoantibodies against these isoforms were able to discriminate individuals with premalignant lesions from healthy individuals, and all of them except p53γ and Δ40p53γ discriminated CRC patients from healthy individuals. Furthermore, CRC patients showed specific induction of a humoral immune response to Δ133p53γ, suggesting that this isoform has the highest diagnostic capacity for CRC patients and individuals with premalignant lesions [65]. This finding is consistent with previous observations showing that this protein is highly dysregulated in CRC and plays an important role in cancer development and progression [40]. In conclusion, p53 isoforms, which differ in their primary sequences and 3D structure, possess differential seroreactivity and diagnostic ability to distinguish CRC patients and individuals with colorectal premalignant lesions from healthy individuals [65].

The expression status of p53 isoform represents the important prognostic value for patients with CRC. Moreover, the expression of specific isoforms can be more relevant in diagnostics than the mutation status of p53. High expression levels of Δ133p53 mRNA in CRC are correlated with shorter DFS and increased tumor aggressiveness [40]. Also, elevated levels of Δ133p53β mRNA are associated with a higher metastatic potential in rectal cancer [39]. However, more extensive research is required to determine whether other p53 isoforms could be used for cancer progression diagnostics. Due to the overexpression and dominant-negative effects in CRC, p53 isoforms present targets whose inhibition could reactivate the p53 pathway and lead to a more effective therapeutic response.

### 2.2. p53 Isoforms in Melanoma

Among different human malignancies, melanoma displays a particularly distinctive pattern of p53 isoform expression and regulation. Although *TP53* is one of the most frequently mutated genes across cancers, mutational inactivation of *TP53* in melanoma is relatively rare, occurring in less than 20% of cases [66,67,68]. Despite expressing wild-type (WT) p53, melanoma cells often exhibit impaired p53 function, characterized by an inability to trigger apoptosis or cell cycle arrest in response to genotoxic stress [68,69,70]. This indicates that mechanisms other than the *TP53* mutation underlie p53 dysfunction in melanoma, including dysregulation of upstream regulators such as MDM2, MDM4, and p14ARF, as well as altered expression of p53 isoforms [71,72,73].

Many studies have investigated the expression and biological functions of p53 family isoforms in melanoma [37,69,74,75,76]. In melanoma cells, compared to fibroblasts, both p53 and small molecular weight isoforms of p53 are aberrantly expressed between the nuclear and cytosolic fraction [69]. For example, Δ40p53 and p53β are upregulated in melanoma cell lines compared to fibroblast and melanocytes, and their expression can be induced by DNA-damaging agents such as cisplatin [69]. These isoforms differentially modulate canonical p53 activity: Δ40p53 acts predominantly as an inhibitor, whereas p53β enhances transcription of apoptosis- and cell cycle-related targets such as p21 and PUMA [38,69,77]. The ratio between specific isoforms and FLp53 is therefore a critical determinant of overall pathway activity [76,78].

Certain N-terminally truncated isoforms, including Δ133p53 and Δ160p53, exhibit pro-oncogenic properties. Elevated Δ133p53α and Δ160p53α levels have been detected in metastatic melanoma compared with normal tissue [37]. The Δ133p53β isoform represents the most prominent pro-oncogenic member of the Δ133p53 variants, being strongly associated with enhanced tumor invasion, metastasis, and overall poor clinical outcomes [37]. Elevated expression of this isoform has also been linked to a higher probability of melanoma recurrence and a shorter time to develop brain metastasis [79]. Furthermore, Δ160p53 isoforms can associate with chromatin to drive proliferation and migration, further contributing to melanoma aggressiveness [76].

In contrast, Δ40p53 demonstrates context-dependent functions. Some studies suggest that Δ40p53 can suppress FLp53 activity and promote survival by activating anti-apoptotic ligands such as netrin-1, correlating with tumor growth and reduced apoptosis in vivo [80]. Conversely, other reports describe the cooperative role of Δ40p53 and p53α in directing cell fate toward apoptosis rather than cell cycle arrest, mediated by increased occupancy of PIDD (p53-induced protein with death domain) promoter and reduced p21 expression [75]. Hence, the biological outcome of Δ40p53 expression depends on its relative abundance and its interaction with FLp53 within the tumor environment.

Aberrant isoform expression also contributes to melanoma resistance to targeted therapies, particularly those inhibiting the MAPK pathway. Vemurafenib-resistant melanoma cells with characteristics of slow-cycling cells exhibit elevated expression of the potentially pro-oncogenic Δ40p53β isoform, along with decreased levels of the tumor-suppressive TAp73β isoform [76,81]. These shifts in isoform balance may facilitate adaptive resistance by reprogramming transcriptional networks controlling apoptosis and cell cycle arrest. Notably, inhibition of p53 itself has been reported to sensitize slow-cycling, therapy-resistant melanoma cells to combined BRAF and MEK inhibition [74].

Clinical data further support a strong association between p53 isoform expression and melanoma progression. Elevated cytoplasmic and nuclear p53β and TAp53 staining correlate with advanced disease stage and reduced survival, whereas lower Δ40p53 expression is linked to less aggressive phenotypes but paradoxically with shorter metastasis-free survival [82]. These findings underscore that the functional state of the p53 pathway in melanoma depends not only on *TP53* mutation status but also on the complex isoform network that modulates its transcriptional and apoptotic functions.

A recent study confirmed the low mutation rate of *TP53* in uveal melanoma (UM) clinical samples and cell lines; however, the response to chemotherapy and proton-beam irradiation was diverse between different cell lines. Analysis of p53 isoform expression revealed the presence of multiple p53 isoforms including Δ160p53α, Δ133p53β, Δ40p53β and p53β. In addition, the correlation between increased expression of Δ40p53α and Δ133p53γ with tumor aggressiveness was found [83].

In summary, melanoma represents a paradigmatic example of a malignancy in which WT p53 is expressed but functionally compromised. This impairment is largely mediated by the differential expression, interaction, and subcellular distribution of multiple p53 family isoforms [38,84,85,86]. The dynamic interplay among these variants dictates whether p53 acts as a tumor suppressor or acquires oncogenic characteristics. Understanding these mechanisms offers novel perspectives on melanoma biology and may pave the way for therapeutic strategies aimed at selectively modulating p53 isoform activity.

Overall, insights from melanoma emphasize the broader relevance of p53 isoform diversity across cancer types. Variants that drive tumor progression and therapy resistance in melanoma, such as Δ133p53, Δ160p53, and Δ40p53, could also exert comparable effects in other malignancies, potentially influencing tumor behavior and treatment outcome. Therefore, melanoma serves as a valuable model for understanding how alternative p53 isoforms fine-tune cell fate decisions and contribute to cancer pathogenesis and therapeutic resistance.

### 2.3. p53 Isoforms in Breast Cancer

Breast cancer, the most frequent malignancy in women, is a heterogeneous disease and is clinically divided into three main types based on the expression of the estrogen receptor (ER), human epidermal growth factor receptor 2 (HER2) and progesterone receptor (PR): ER-positive (ER+), HER2 amplified, and triple-negative breast cancer (TNBC, lacking the expression of ER, PR and HER2) [87]. The fact that *TP53* somatic mutations in breast cancer are less common (present in 25–30% of breast cancers) compared to most cancer types implies that the p53 activity could be modulated by different mechanisms, involving the p53 isoform network [88]. Thus, in breast cancer, the *TP53* mutational status has not proven to be efficient biomarker, and its gene expression signature is shown to be the more relevant predictor of chemotherapy response and prognosis [89,90,91].

Avery-Kiejda and colleagues analyzed relative mRNA expression of the p53 isoforms in several breast cancer cell lines, tumor samples and matched normal tissue. The most prominent finding was increased expression of Δ40p53 isoform in tumor tissues compared to normal samples, correlating with the TNBC, which is the most aggressive breast cancer subtype. In contrast, higher expression of p53β isoform was associated with smaller tumor size and increased DFS in patients irrespective of the *TP53* mutation status. Interestingly, patients with low expression of p53β and mutation in *TP53* had the worst prognosis [92]. However, in contrast to isoform expression analysis at the mRNA level, a study of protein isoform expression by immunohistochemistry using isoform-specific antibodies revealed that high cytoplasmic level of p53β isoform is related to worse prognosis independently of the *TP53* mutational status [93]. Furthermore, in the previously mentioned study by Avery-Kiejda and coworkers, the lowest expression level was attributed to p53γ [92], the isoform that was previously reported to have a beneficial effect on the prognosis of breast cancer patients carrying the *TP53* mutation [94]. Namely, the presence of the p53γ expression in tumors with mutated *TP53* correlated with low recurrence risk and survival comparable to patients expressing WT p53 [94]. Interestingly, these findings can corroborate the results of Avery-Kiejda et al. by showing that higher p53γ isoform expression positively correlated with lower tumor grade [92].

The same group further investigated the role of the Δ40p53 isoform in breast cancer, more precisely, the importance of the Δ40p53:p53α ratio in tumorigenesis. They found that increased Δ40p53:p53α ratio correlated with impaired cell cycle regulation (decreased G1 arrest, increased G2 arrest), decreased sensitivity to chemotherapeutics, and inhibition of apoptosis-related genes [95].

Furthermore, the same group analyzed the relation between Δ40p53 and cancer stem cell regulation and its impact on the response of breast cancer to therapy. High levels of Δ40p53 correlated with downregulation of differentiation-related genes and upregulation of genes related to stem cell regulation in invasive ductal carcinoma [32]. Moreover, endogenously expressed Δ40p53 co-localized with SOX2, NANOG, and OCT4 in MCF-7 and ZR75-1 breast cancer cell lines, and with SOX2 in ZR75-1 cell spheroids [32]. These results were corroborated by the increased expression of *SOX2*, *OCT4*, and *NANOG* obtained by single-cell and bulk RT-qPCR in MCF-7 cells overexpressing Δ40p53. Increased level of *ZEB1* transcription factor and *CDH1* (encoding E-cadherin) were also found in cells overexpressing Δ40p53. Furthermore, compared to parental cells, the cells with high level of Δ40p53 showed stronger mammosphere and colony-formation abilities, as well as downregulation of miRs known to act as potential repressors of stemness such as miR-145, miR-200a, and miR-200b. Injection of MCF-7 cells with high Δ40p53:p53 ratio into NSG (NOD scid gamma) mice resulted in augmented tumor growth, Ki67 and Sox2 expression, and blood microvessel areas, and decreased sensitivity to doxorubicin compared to tumors derived from parental cells. The efficacy of doxorubicin was enhanced upon Δ40p53 silencing or co-treatment with MELK inhibitor OTSSP167, supporting targeting of Δ40p53 as a potential therapeutic approach to increase the effect of standard therapies [32].

As Δ40p53, the Δ133p53β isoform can also promote stemness potential of MCF-7 cells by endorsing mammosphere formation and positively regulating expression of *SOX2*, *OCT3/4*, and *NANOG*, as well as proportion of CD44^+^/CD24^−^ cells. In addition, chemotherapy treatment (etoposide) upregulated the expression of Δ133p53 and caused activation of the aforementioned key pluripotency/reprogramming regulators [31]. Gadea and coworkers have analyzed the mRNA expression of different Δ133p53 C-terminal splice variants (Δ133p53α, β, and γ) by nested RT-PCR in a cohort of breast cancers of different subtypes. Interestingly, the Δ133p53β isoform was more frequently expressed in tumors harboring mutant p53 compared to tumors carrying WT p53. They also showed that the Δ133p53β isoform promotes breast cancer cell invasion independently of *TP53* mutational status and augments the risk of cancer recurrence leading to decreased overall survival (OS) [26,96]. In addition, increased expression of Δ133p53β isoform was found in brain metastases compared to primary breast tumors and associated with reduced time necessary for the primary tumor to metastasize to the brain. Notably, the expression of Δ133p53β had no significant impact on the OS after diagnosis of brain metastasis [79]. However, although it is known that mutant p53 favors metastasis, no correlation was found between the presence of mutant p53 and Δ133p53β.

Another group of researchers reported that the Δ133p53 isoform upregulates interferon-gamma (IFN-γ) signaling which is related to better patient outcome [97]. They analyzed the relation between main signaling pathways and the expression of p53 family members in several groups of breast cancers stratified by different ER and *TP53* mutation status. Increased Δ133p53 mRNA level and activity of the IFN-γ signaling was specifically found in ER-positive (ER+) tumors harboring *TP53* mutations [97].

In addition, reciprocal regulation of p68 RNA helicase and Δ133p53α has been identified in primary breast cancers participating in complex feedback mechanisms affecting p53-dependent DNA damage response, particularly regulation of the *CDKN1A* (p21) promoter [98].

### 2.4. p53 Isoforms in Ovarian Cancer

Ovarian cancer represents one of the deadliest cancers among women worldwide and, in spite of its predominantly epithelial origin, is highly heterogeneous regarding its histological and molecular characteristics reflecting diverse response to different therapies. Epithelial ovarian cancer is commonly divided into five major subtypes: clear cell (CCOC), endometrioid (ENOC), mucinous (MOC), low-grade serous (LGSOC) and high-grade serous ovarian carcinoma (HGSOC). These subtypes exhibit different genetic profiles including different prevalence of *TP53* mutations. Whereas the *TP53* gene is almost ubiquitously mutated in HGSOC, other subtypes exhibit a lower frequency of *TP53* mutations (in LGSOC they are extremely rare) [99].

Despite the high frequency of *TP53* mutations in HGSOC, the most frequent and severe ovarian cancer type, *TP53* mutational status has not proven to be an efficient prognostic marker in advanced serous ovarian carcinoma. In addition, the initial studies of the p53 isoforms’ mRNA expression revealed no significant differences in Δ40p53 and Δ133p53 levels between low- and high-grade ovarian cancers [100]. On the other hand, a study by Hofstetter and colleagues demonstrated a correlation between increased Δ133p53 expression and better recurrence-free (RFS) and OS in patients carrying the *TP53* mutation. These findings indicate that Δ133p53 may interact with mutant p53, thereby abolishing the negative effects of mutant p53 [101]. The authors also reported the relation between low Δ133p53 levels and resistance to platinum-based therapy. In contrast, increased expression of the Δ40p53 isoform correlated with increased RFS in patients with WT p53 and lower tumor grade.

The same group further investigated the gene expression of Δ40p53, Δ133p53 and FLp53 in a subset of mucinous, endometrioid and serous ovarian carcinoma samples compared to normal ovarian tissues [102]. They found that Δ133p53 expression was lowest in endometrioid ovarian cancer, whereas the expression levels of FLp53 and Δ40p53 did not differ significantly among the examined cancer subtypes. In addition, Δ133p53 expression was significantly lower in endometrioid cancer tissues compared to normal tissue samples. In mucinous ovarian cancer specimens, the level of Δ40p53 was significantly increased compared to normal tissues and associated with improved RFS [102].

Bischof and colleagues investigated the alterations in the p53 pathway in HGSOC, more precisely the predictive and prognostic effects of the p53 isoforms’ gene expression (total p53, total Δ133p53, p53β, and p53γ) and *TP53* mutations in tumor tissues of chemotherapy responders and non-responders [103]. *TP53* loss-of-function and hotspot mutations are shown to be predictive of tumor characteristics and disease progression, which is in accordance with the previous research [104]. Although no significant difference in expression was found between chemosensitive and chemoresistant subgroups, a positive correlation was found between improved OS and high levels of total Δ133p53, indicating that it could be a promising predictive biomarker in HGSOC [103].

Demethylation/methylation processes could be one of the potential mechanisms of modulating the p53 biological functions by affecting the expression of short or long p53 isoforms [105]. It was found that the *TP53* promoter is methylated in 51.5% of ovarian carcinoma specimen, compared to 29.7% of healthy ovaries [106]. A recent study revealed methylation of introns 3 and 4 of *TP53*, whereby the methylation status of the intron 4 could be of particular interest as it is the location of the alternative promoter P2 [105].

### 2.5. p53 Isoforms in Endometrial (Uterine) Carcinoma

In endometrial carcinoma, the most frequent gynecological cancer, the p53 overexpression is associated with disease recurrence, while *TP53* mutations correlate with the aggressive phenotype [107]. In uterine serous carcinoma, the aggressive subtype of endometrial cancer that resembles molecularly HGSOC and basal-like breast cancers, the *TP53* gene, is frequently mutated. Although the expression analysis revealed that the most abundant isoform was Δ133p53, no correlation with clinical parameters has been found. On the other hand, higher relative expression of p53γ positively correlated with shorter progression-free survival [108]. In addition, in endometrial carcinoma cell lines, FLp53 and Δ40p53 are the predominant isoforms, with Δ40p53 mainly detected in the cytoplasm in the form of amyloid aggregates that can modulate p53 functions [109]. Intriguingly, WT p53 aggregation has also been reported in HGSOC cells exhibiting cancer stem cell properties, which is associated with p53 loss-of-function and platinum resistance [110].

### 2.6. p53 Isoforms in Prostate Cancer

Prostate cancer (PC) is the second-most-common cancer in men and, similar to most cancers, represents a genetic disease characterized by multiple genomic alterations, including point mutations and microsatellite variations. Compared to other cancers, PC is known for having high prevalence of chromosomal alterations that often result in gene-fusion events [111]. The frequency of genetic alterations between primary and advanced PC is most evident for *TP53* mutations that vary around 10% to up to 50%, respectively [112,113]. Specific *TP53* mutations are associated with PC progression, recurrence, therapy resistance, and poor outcome [112,114,115]. The key driver of PC initiation and progression is chronic inflammation [116] which is shown to be associated with the Δ133p53 isoforms [27,35,36]; therefore, it is understandable that its role has been investigated in PC [117].

Kazantseva and coauthors investigated the potential correlation between p53 isoforms, inflammation and PC progression using a cohort of 122 tumor samples and 3 non-tumor negative controls [117]. The authors reported increased expression of Δ133p53β in PC compared to control samples. They noticed that increased Δ133p53β levels were associated with high proliferative index, increased CD3+ T-cells, PD-1 on infiltrating T-cells, PD-L1 on cancer cells, CD163 and CSF1R-positive macrophages, higher risk for developing aggressive cancer and poor patient outcome. Interestingly, increased levels of Δ40p53 and p53α were associated with a more favorable prognosis. On the protein level, the elevated Δ133p53β in cancer tissue was attributed to cancer cells but not immune cells. To determine the molecular insights of Δ133p53β-mediated PC progression, the authors performed RNA sequencing on 12 tumor samples and four healthy tissues. Gene enrichment analysis showed that Δ133p53β is involved in immune regulation, PD-1 signaling, cell invasion and angiogenesis. These findings were supported by in vitro experiments, where transiently expressed Δ133p53β in PC3 cells significantly increased the expression of *STAT6* and *CXCR6*, whereas Δ133p53 knockdown resulted in a significant reduction of *IL6ST*, *STAT6* and *CXCR6* expression in 22Rv1 cells. Further, the authors determined that hypoxia stimulates Δ133p53 expression in PC cells. Increased Δ133p53 expression was observed not only under hypoxia in 22Rv1 cells harboring WT p53 DBD but also a heterozygous mutation at the C-terminus WT/Q331R, which affects the *TP53β* splicing. On the other hand, hypoxia did not influence Δ133p53 expression in DU145 cells with mutations in DBD, P223L/V274F. Hypoxia moderately increased the levels of FLp53 and Δ40p53 in both cell lines tested. The expression of *VEGFA* gene, a biomarker of hypoxia and regulator of angiogenesis, was increased in 22Rv1 cells which implies that WT p53 enhances hypoxic conditions. To prove that the p53 isoforms can directly regulate hypoxia-related genes, the authors transiently expressed Δ133p53β and observed increased *VEGFB* expression in PC3 cells, while Δ133p53 knockdown reduced the expression of both *VEGFA* and *VEGFB* in 22Rv1 cells. Similar results were obtained using the H1299 lung cancer cell line where increased expression of Δ133p53β upregulated *VEGFA*, *VEGFB*, *KDR* and *EGFR* genes.

Interestingly, the authors also reported that the *CD274* expression, which encodes PD-L1 immune regulator, is directly regulated by Δ133p53β. Using H1299 cells, Δ133p53β overexpression increased *CD274* levels compared to cells that overexpressed Δ133p53α or control cells. Similar results were obtained using PC cells, where PC3 cells transfected with Δ133p53β expressing plasmid showed increased levels of *CD274*, while knockdown of Δ133p53 in 22Rv1 reduced the levels of *CD274* [117]. In conclusion, Δ133p53β is upregulated in PC and is associated with inflammation, angiogenesis, and immunosuppressive conditions, regulates the levels of hypoxia-related genes and genes involved in immune regulation, and promotes aggressive features of PC. Further research is needed to unravel additional biological functions of Δ133p53β that lead to PC aggressiveness.

### 2.7. p53 Isoforms in Lung Cancer

Lung cancer is classified into non-small-cell (NSCLC) and small-cell lung cancer (SCLC) based on cell origin and histological characteristics [118]. NSCLC accounts for approximately 85% of cases, and its prevalent subtypes include adenocarcinoma and squamous cell carcinoma. Smoking is the leading cause of NSCLC, but non-smokers can also be affected due to their genetics and environment. The remaining 15% of lung cancer cases refer to SCLC with over 95% of cases associated with tobacco use. Smoking-related genetic changes commonly include mutations in tumor-suppressor genes *RB1* and *TP53*, contributing to rapid metastatic spread, which makes SCLC a more aggressive cancer type compared to NSCLC. Although the interplay between p53 family members, mostly focusing on compensatory mechanisms of p73 in response to p53 loss, has been investigated in SCLC, the role of p53 isoforms in SCLC still needs to be elucidated [119].

To our knowledge, only one study described the status of p53 isoforms in lung cancer, mostly focusing on the Δ133p53 isoform in NSCLC [42]. The expression of Δ133p53 was determined in 17 lung cancer tissue samples, including both squamous cell lung cancer and lung adenocarcinoma as well as corresponding adjacent healthy tissue. A significant overexpression of Δ133p53 mRNA was observed in tumor tissues compared to matching healthy tissue. Additionally, increased expression of MDM2 and the FLp53 as well as reduced expression of p21 was reported in cancer samples compared to healthy tissue. Although not statistically significant, the reduced expression of p21 correlated with elevated Δ133p53 levels. Interestingly, smoking status correlated with the increased expression of Δ133p53, with lowest expression of Δ133p53 observed in non-smokers, which increased from intermediate to heavy smokers. This suggests that smoking could be associated with Δ133p53 overexpression; therefore, the exploration of Δ133p53 in smoking-related lung cancers, such as SCLC, would be of great importance. Next, the authors tested whether increased expression of Δ133p53 correlates with higher protein levels in cancer tissue compared to healthy controls. Although there was no strict correlation between transcript and protein expression of Δ133p53, both Δ133p53 and Δ40p53 isoforms were elevated in some tumor samples compared to corresponding healthy controls. Furthermore, C-terminus-truncated p53β and p53γ were detectable in most samples. In the majority of cases tested, the p21 protein had lower levels in tumor samples. Overall, these findings imply that elevated levels of Δ133p53 mRNA could be a potential biomarker of NSCLC [42]. Further studies are needed to elucidate the role of truncated p53 isoforms in dysregulated cell cycles or other biological processes involved in NSCLC.

### 2.8. p53 Isoforms in Head and Neck Squamous Cell Carcinoma

Similar to lung cancer, mutations in *TP53* are the most frequent somatic genetic alterations in head and neck squamous cell carcinoma (HNSCC), causing dysregulation of the p53 pathway [120]. Besides the loss-of-function, certain mutant p53 proteins can exhibit a dominant-negative effect by forming heterotetramers with WT p53, while acquiring oncogenic properties that contribute to tumorigenesis and cancer progression. The p53 mutated HNSCC are associated with poorer clinical outcomes, such as cancer resistance to chemo- or radiotherapy and shorter HNSCC patient survival. The functional p53 pathway may also be disrupted due to dysregulated p53 isoforms activity, and several studies have investigated the expression of p53 isoforms in HNSCC and related premalignant lesions.

Boldrup and coauthors have shown that both p53β and Δ133p53 can be determined at the mRNA level both in HNSCC and corresponding normal tissues with p53β being the most readily identifiable isoform in HNSCC. However, due to methodology limitations which were related to the antibodies’ specificity, p53β and Δ133p53 could not be reliably detected in the HNSCC tissue samples at the protein level [121].

In a recently reported study, immunohistochemical analysis (IHC) was performed on a series of head–neck lesions that arose during malignant progression, from benign papillomas (PA) along the inverted papilloma (IPA) to malignant squamous cell carcinomas (HNSCC) with the aim to evaluate possible changes in expression of p53 and its isoforms related to HNSCC development and progression. The authors observed higher expression of p53 isoforms in IPA and HNSCC compared to PA. Interestingly, IPA lesions showed the highest immunoreactivity of p53 isoforms, suggesting the potential role of p53 isoforms in the development of local aggressiveness. One could hypothesize that specific p53 isoforms, such as p53β or Δ133p53, might drive invasion of epithelial cells in IPA lesions [122].

The expression of p53 isoforms has also been tested in oral lichen planus (OLP). OLP is a chronic inflammatory disease of skin and mucosa that is classified as premalignant lesions by WHO, and can develop to HNSCC with prevalent transformation rates around 1–2%. Interestingly, the gene expression levels of both p53β and Δ133p53 isoforms are shown to be lower in OLP compared to HNSCC and normal tissue. Additionally, the isoforms of p53 family members p63, TAp63 and ΔNp63, are also shown to be downregulated in OLP compared to HNSCC samples [123].

### 2.9. p53 Isoforms in Gastric Cancer

Gastric cancer (GC) is a highly aggressive cancer and due to its usually asymptomatic early stage it is frequently diagnosed at an advanced stage when the tumor is already unresectable. The gold standard is still platinum-based chemotherapy or radiotherapy, but with moderate efficiency for advanced, metastatic stage disease. Although prognosis for GC patients are frequently poor with a median survival of 12 months, perioperative or adjuvant chemotherapy increase the chance of patient survival [124,125].

The majority of GC is associated with viral (Epstein–Barr virus) or bacterial (*Helicobacter pylori*) infections, while a small minority is related to hereditary mutations [126]. The progression of GC is associated with the *TP53* mutations, which can initiate transformation of stomach epithelial to cancer cells and therefore are considered early, founding events in GC development. The *TP53* mutations can be induced by *Helicobacter pylori* (*H. pylori*) infection and are shown to be more frequently present in specific types of GC [127,128,129]. The involvement of specific p53 isoforms in GC progression and the changes in expression status upon treatment with different therapy agents have been investigated in several studies by Ji and coauthors. In one of their studies, the authors determined the mRNA expression of p53β and Δ133p53 in tissues associated with gastric malignant progression. They observed a specific p53 expression signature which correlates with gastric carcinogenesis and includes increase in ∆133p53 and decrease in p53β mRNA from superficial to atrophic gastritis, as well as paracancerous lesions up to gastric adenocarcinoma [41]. Using GC cell lines with different p53 status (WT (MKN45 line), whether mutated (SGC-7901 line) or deleted (KATOIII line) p53), Ji and coauthors determined the mRNA level of both p53β and Δ133p53 with or without cisplatin treatment. Interestingly, both p53β and Δ133p53 were detected in WT p53, while only p53β was expressed in p53 mutated cell line. Neither p53β nor Δ133p53 were expressed on mRNA level in KATOIII cell line. Cisplatin treatment significantly inhibited growth of both MKN45 and SGC-7901 cancer cell lines, increasing the expression of p53β only in the MKN45 cell line, while the level of p53β remained unaffected in the SGC-7901 cell line. In addition, the expression of p53β positively correlated with the expression of both p53 and BAX in the cisplatin-treated MKN45 cell line, while the expression of p53β positively correlated with the expression of p53 but not of BAX in the cisplatin-treated SGC-7901 cell line. The results of this study suggest that the p53β isoform has different roles in GC cells with different p53 mutation backgrounds [130]. Ji and coauthors also reported that the treatment with recombinant mutated human tumor necrosis factor (rmhTNF) causes growth inhibition of MKN45 cell lines with synergistic effects when combined with cisplatin. Although rmhTNF treatment alone did not influence p53β or Bcl-2 expression levels, the upregulation of p53β and downregulation of Bcl-2 were observed after combined rmhTNF and cisplatin treatment. Based on their results, the authors concluded that the inhibitory effect of cisplatin can be enhanced by the rmhTNF treatment via unknown mechanisms that includes p53β in MKN45 cells [131]. Interestingly, rmhTNF treatment alone causes downregulation of ∆133p53 in a dose-dependent manner in MKN45 cells [132]. The influence of NF-κB inhibitor pyrrolidine dithiocarbamate (PDTC) on p53 isoform expression and growth inhibition of MKN45 cell line was also tested. Significant downregulation of ∆133p53 was observed in a dose-dependent manner by PDTC alone or in combination with cisplatin. On the other hand, p53β expression was not affected by PDTC treatment [133].

As already mentioned, *H. pylori* infection can actively inhibit p53 tumor suppression pathway probably by facilitating activation of MDM2-dependent p53 degradation [134,135]. Interestingly, *H. pylori* infection can also alter the expression levels of N-terminally truncated p53 isoforms, which are known to inhibit both p53 and p73 activities [84]. In more detail, Wei and coauthors have shown that infection of gastric epithelial cells (AGS and SNU-1) with *H. pylori* increased the expression of Δ133p53 and Δ160p53 isoforms. Additionally, the authors observed that downregulation of Δ133p53 in *H. pylori*-infected cells resulted in increased expression of p53-targets, p21 and NOXA. Δ133p53 was also shown to modulate the transcriptional activity of both p53 and p73, enhance NF-κB activity by influencing the expression of NF-κB targets such as IL-6, Bcl-2, and IL-8, and increase the survival of *H. pylori*-infected cells. Notably, downregulation of c-Jun, an important component of activator protein-1 (AP-1) transcription factor, inhibited Δ133p53 expression at both mRNA and protein levels, suggesting that Δ133p53 transcription is regulated via AP-1 in *H. pylori*-infected cells [55].

### 2.10. p53 Isoforms in Hepatocellular Carcinoma

Loss of p53 tumor suppressor functions is a key event in hepatocarcinogenesis, contributing to the development of hepatocellular carcinoma (HCC), one of the most common malignancies worldwide [136,137,138]. The importance of p53 in HCC development has been demonstrated by reactivation of endogenous p53 in a murine liver carcinoma model, which induced senescence and suppressed tumor cell growth [139]. Although the role of Δ40p53α in HCC development is not completely understood, Ota and colleagues showed that Δ40p53α acts as a tumor suppressor. Notably, *TP53*+/Δ40 HepG2 cells expressing endogenous Δ40p53α did not increase caspase activity or annexin V- and PI-positive cell population, indicating that its tumor-suppressor activity is not mediated through apoptosis [140]. Ectopic Δ40p53α expression suppressed colony formation in both WT p53 expressing and p53-null HCC cells, suggesting that FLp53 status is irrelevant for Δ40p53α tumor-suppressor activity. Moreover, Δ40p53α exerts stronger tumor-suppressor activity than canonical p53, as it induced senescence, upregulated p53 target gene expression, and inhibited clonogenicity in the presence of p53α. When HepG2 and PLC/PRF/5 cells were treated with doxorubicin, higher expression levels of Δ40p53α were observed, indicating that it may have a promising anti-tumor role in HCC. It appears that Δ40p53α also plays a crucial role in regulating senescence in HCC cells. An increased number of senescence-associated β-galactosidase (SA-β-gal)-positive cells were observed among the HepG2 cells expressing Δ40p53α compared to the control cell clones. These cells also had higher mRNA and protein levels of p21 and other p53-inducible genes, including MDM2 and FAS [140]. Interestingly, Δ40p53α-induced anti-tumor activity was significantly reduced in cells expressing hotspot mutant Δ40p53α-R175H, which lacks transcriptional activity. Cells expressing this mutant formed fewer colonies, exhibited increased SA-β-gal positive staining, and showed no changes in mRNA levels of *p21*, *MDM2* and *FAS*, indicating that the transcriptional activity of Δ40p53α is associated with its tumor-suppressor functions. Moreover, Δ40p53-R175H cells showed upregulation of CCNB1 and/or IL-8, proteins that lead to a senescence-associated secretory phenotype (SASP), providing evidence that upregulation of p21 is not the only mechanism by which Δ40p53 induces cellular senescence [140]. Previously published studies have shown that Δ40p53α regulates p53-inducible gene expression in both positive and negative way [85]. In line with this, Ota et al. demonstrated that Δ40p53α increases the protein half-life of canonical p53 and broadened the FLp53-induced anti-tumor activity, suggesting that Δ40p53α could positively regulate FLp53 activity. In conclusion, Δ40p53α exerts tumor-suppressor activity and promotes cellular senescence in HCC cells by upregulating p53 target gene expression [140]. However, further studies are needed to clarify the role of Δ40p53α and its mutants in the pathogenesis of HCC, as well as their potential therapeutic relevance in HCC.

### 2.11. p53 Isoforms in Cholangiocarcinoma

Cholangiocarcinoma (CCA), a type of liver cancer alongside HCC, is a malignant tumor arising from the bile duct epithelium. CCA is often difficult to diagnose as it is asymptomatic in the early stages and there are no specific biomarkers for detection [141,142]. *TP53* is one of the most frequently mutated genes in CCA associated with river fluke *Opisthorchis viverrini* (OV) infection, indicating its role in CCA carcinogenesis [143]. Puetkasichonpasutha et al. showed that p53 protein is elevated in most CCA tissue samples and inversely correlated with *WIP1* (wild-type p53-induced phosphatase 1) mRNA expression, which is a p53 target and negative regulator [144]. In addition, SIRT1 deacetylates p53 in liver tumors, leading to inhibition of the cell cycle arrest [145]. Elevated levels of mutant p53 protein have been detected in CCA tissues, with the majority of missense, hotspot mutations located in DBD [146,147]. These mutations reduce therapeutic efficacy and increase resistance to chemotherapy in CCA patients [148]. In addition to mutant p53, ΔNp53 was simultaneously detected in CCA tissues, suggesting another possible mechanism for p53 inactivation. Interestingly, patients expressing the ΔNp53 isoform had better OS compared to those harboring mutant p53, indicating that ΔNp53 did not completely inhibit p53 transcriptional activity, while mutant p53 was completely non-functional [43]. Additional studies are needed to fully elucidate the distinct roles of mutant p53 and ΔNp53 in CCA. Furthermore, studies have shown that a high Δ133p53/p53 mRNA expression ratio correlates with poor OS in CCA patients [43]. Specifically, the Δ133p53 isoform is associated with resistance to 5-fluorouracil (5-FU) in CCA cell lines KKU-M214R and KKU-M139R. In 5-FU-resistant tumor tissues and CCA cells, Δ133p53 was upregulated in a dose-dependent manner. Suppression of Δ133p53 restored sensitivity to 5-FU and promoted apoptosis through upregulation of the pro-apoptotic protein BAX and downregulation of the anti-apoptotic protein Bcl-2 [30]. These findings suggest that Δ133p53 may act as a dominant-negative regulator of WT p53, inhibiting cell cycle arrest and apoptosis, and eventually contributing to CCA. Additionally, increased p73 expression was observed upon Δ133p53 silencing in CCA cell lines. Altogether, these results suggest that the Δ133p53/p53 ratio can serve as a prognostic biomarker and that ∆133p53 may be a potential target and modulator of chemosensitivity in CCA [30].

### 2.12. p53 Isoforms in Renal Cancer

p53 is mutated in fewer than 10% of renal cell carcinomas (RCCs) [149]. Although only a small number of studies have investigated p53 isoforms in RCC, the available evidence suggests that they may play important roles in tumor progression, patient survival, and response to therapy.

In the study by Song and colleagues, the Δ133p53 isoform was detected exclusively in tumors overexpressing p53β mRNA, which also correlated with tumor stage. Western blot analysis further confirmed the presence of these isoforms at protein level [150]. Another study on RCC patient samples demonstrated upregulation of three long isoforms, p53α, p53β, and p53γ at the mRNA level compared to healthy renal tissues. Interestingly, Δ133p53 isoform expression was downregulated in the early tumor stages, whereas in advanced stages, the expression of Δ40p53α and Δ40p53γ was increased. Furthermore, in vitro treatment of ClearCa-3 renal cancer cells with topotecan induced alterations in p53 isoform expression [151]. In a large cohort of patients with clear cell RCC, expression levels of p53α, p53β, and p53γ were examined. While p53α and p53γ levels remained relatively stable across tumor stages, p53β expression progressively decreased with advancing tumor stage. Consistent with these findings, high p53β expression was shown to be an independent predictor of improved OS and RFS, regardless of *TP53* mutational status, potentially through enhancing tumor cell apoptosis [152]. In our study of RCC patient samples, p53, Δ40p53, and Δ133p53 isoforms were identified in tumor tissues, with a significant downregulation of Δ133p53 observed in tumors harboring WT p53 compared with adjacent normal tissue. In contrast, tumors harboring mutant (mt) p53 exhibited significantly higher expression of p53, Δ40p53, and particularly Δ133p53 isoforms. Elevated mRNA expression of p53 and Δ40p53 was associated with larger tumor size and renal capsular invasion. Notably, younger patients displayed higher expression levels of the Δ133p53 isoform [153]. Clear cell RCCs expressing Δ133p53α mRNA were found to be smaller in size while isoforms ∆40p53α and ∆40p53γ could not be detected [154].

### 2.13. p53 Isoforms in Glioblastoma

Glioblastoma (GBM) is a highly aggressive adult brain tumor characterized by rapid growth, resistance to therapy, and poor clinical outcome. Dysregulation of p53 signaling pathways plays a central role in GBM pathogenesis, involving p53 mutations, functional inactivation of p53, and contribution of distinct p53 isoforms [155]. Standard post-surgical therapies, including radiotherapy and chemotherapy with temozolomide, induce cellular senescence in a p53-dependent manner [155,156,157]. Takahashi and colleagues reported Δ40p53 expression in the majority of examined GBM xenografts and patient samples [158]. In contrast, FLp53 (TAp53) expression was more prominent in xenografts propagated in nude mice than in primary tumors. This study also points to the similarity in TAp53 and Δ40p53 expression patterns between GBM and neural stem progenitor cells [158]. However, subsequent studies more consistently emphasized the functional role of Δ133p53 isoforms in GBM [28,159,160]. Isoform profiling of human GBM cell line U87 demonstrated the expression of TAp53α/γ and Δ133p53α/β [28]. Increased Δ133p53β expression has been observed in a subset of GBMs telomerase-like activity tumors (TELM), in which the precise telomere maintenance mechanism remains undefined [159]. These tumors exhibit high macrophage infiltration, reduced sensitivity to temozolomide, and poor prognosis. Δ133p53β expression is enriched in malignant cells located in hypoxic tumor regions as well as in stromal cells surrounding blood vessels, where an increased proportion of PDL1- and CSF1R-positive cells indicates an immunosuppressive microenvironment. Mechanistically, expression of the murine Δ133p53β ortholog, Δ122p53, in mouse fibroblasts upregulates expression of CCL2, a key chemokine involved in recruiting macrophages and promoting the establishment of an immunosuppressive environment [159]. Further evidence implicates Δ133p53α in the regulation of angiogenesis [28]. siRNA-mediated downregulation of Δ133p53α in U87 cells induces upregulation of TAp53 and shifts gene expression towards an anti-angiogenic secretome, thereby inhibiting endothelial cell migration and tubulation in vitro as well as angiogenesis and tumor growth in vivo. In contrast, TAp53 downregulation does not alter Δ133p53α expression, but enhances pro-angiogenic properties of GBM. The pro-angiogenic role of Δ133p53α is mediated through VEGF-independent mechanisms involving factors such as ANG, HGF, and ANGPTL4 [28]. Available data indicate that Δ133p53α reduces expression of EMT markers and invasiveness, without significantly affecting proliferation and cell growth. On the contrary, Δ133p53α harboring the common p53 mutation R273H (Δ133p53α R273H) promotes proliferation, EMT marker expression, and invasiveness in both 2D cultures and 3D spheroid GBM models. Although both WT and mutated Δ133p53α inhibit apoptosis, only mutated Δ133p53α fails to prevent genomic instability due to impaired expression of the key homologous recombination protein, RAD51. Conversely, Δ133p53α promotes RAD51 expression and thus retains the DNA-repair function. Moreover, Δ133p53α R273H induces expression of IDO1 and IL4i1 and activates the AHR receptor, promoting immunosuppressive response [160]. In the therapeutic context, both murine Δ122p53 and human Δ133p53α confer increased resistance to temozolomide and prevent induction of cellular senescence through downregulation of senescence-associated genes such as *CDKN1A* [159,160]. Conversely, Δ133p53α R273H prevents senescence following radiotherapy [160]. In summary, accumulating evidence emphasizes the importance of Δ133p53 isoforms in promoting angiogenesis, immune evasion and therapeutic resistance of GBM. The presence of mutant Δ133p53 R273H further exacerbates the malignant phenotype, driving tumor progression and enhancing aggressiveness.

### 2.14. p53 Isoforms in Sarcoma

Only a few studies have investigated p53 isoforms in sarcomas. A study investigating the impact of p53β on chemosensitivity to doxorubicin in *TP53*-null Saos-2 cell line demonstrated that transient transfection with p53β resulted in a significant reduction in cell proliferation. In contrast, transfection with p53γ or FLp53 did not produce this effect [161]. A mechanistic paper investigating the role of Δ133p53α in response to DNA damage in different cell models noted that Δ133p53 gene expression was significantly induced upon doxorubicin treatment in U2OS osteosarcoma cell line (WT p53) [162]. Several studies have investigated Δ122p53, a murine homolog of Δ133p53. One study showed that sarcomas from Δ122p53 homozygous mice have invasive and metastatic properties. In human sarcoma cells Saos2 Δ133p53 also showed malignant properties, promoting cell invasion in vitro, similar to the GOF phenotypes associated with mutant p53. In a B16 mouse melanoma metastatic model, Δ122p53 accelerated the formation of lung metastases [27].

Ewing sarcoma (EWS) expresses high levels of Δ133p53 isoforms, which induces Hepatocyte growth factor (HGF) secretion, promoting tumor growth and metastasis in mice. Targeting EWS with an HGF receptor-neutralizing antibody (AMG102) enhanced leukocyte infiltration and, in combination with GD2-specific CAR T-cell therapy, synergistically inhibited both primary tumor growth and metastatic spread [163].

### 2.15. p53 Isoforms in Leukemia

*TP53* mutations are present in approximately 5–10% of patients with de novo acute myeloid leukemia (AML), occurring more frequently in elderly individuals and in those with therapy-related AML [164]. Although *TP53* mutations are relatively rare in AML compared to other tumors, the function of WT p53 is often inactivated through various mechanisms [165]. Only a small number of studies have explored the role of p53 isoforms in AML, mostly using 1D and 2D gel immunoblots. Results demonstrated the presence of two main forms of p53: the full length (including p53α, p53β, and p53γ) and a group of smaller-weight isoforms referred to as Δp53. In untreated patients, Δp53 are more expressed than p53α, but chemotherapy with idarubicin and cytarabine markedly induced p53α [166]. Another study introducing a novel automated bioinformatics analysis of 2D gel blots of AML samples detected p53-α, p53-δ, and sub-δ isoforms [167]. Further studies have identified the FLp53, p53β, and p53γ isoforms in AML patients. Surprisingly, expression of the FLp53 isoform was associated with adverse prognosis, whereas the p53β and p53γ isoforms correlated with long-term survival and favorable prognosis after chemotherapy [168]. Differentiation therapy with valproic acid (VPA) significantly affected p53 isoform expression, more precisely downregulation of p53β/γ and upregulation of FLp53. Expression of FLp53 positively correlated with VPA sensitivity and FAB classification of AML, whereas p53β/γ isoforms showed negative correlations [169].

Research on p53 isoforms in other types of leukemia remains limited. In B-cell chronic lymphocytic leukemia (CLL), one study reported an increased ratio of FLp53 to its β and γ isoforms compared with normal B cells. Notably, higher ratios were associated with inferior clinical outcomes, including shorter treatment-free survival and OS [170]. In B-cell precursor acute lymphoblastic leukemia (BCP-ALL), a separate study identified a complex pattern of N-terminally truncated p53 isoforms that varied with the disease course. Specifically, primary BCP-ALL samples showed high expression of TAp53, whereas relapsed samples were characterized by elevated Δ40p53. In contrast, Δ133p53 and p53β were increased in both primary and relapsed disease [171].

Alterations in the expression of p53 isoforms may play a significant role in the functional dysregulation of the p53 pathway across different types of leukemia, potentially influencing disease progression and therapeutic response.

Summarized biological functions of specific p53 isoforms in different cancer entities are comprised in Table 1.

## 3. Targeting of p53 Isoforms

p53 isoforms are considered attractive therapeutic targets due to their differential expression in tumor vs. normal cells, their interacting partners, and regulatory expression mechanisms. As individual isoforms are differentially expressed in various cancers, factors affecting the ratio of p53 isoforms may have therapeutic potential. Moreover, both direct and indirect modulation of p53 isoform expression could impact cancer treatment. It has been reported that translation initiation of p53α and Δ40p53 is driven by an IRES (internal ribosome entry site) present in the 5′ terminal region [172]. Most IRESs are regulated by IRES trans-acting factors (ITAFs), which affect the IRES structural conformation, thereby modifying their recognition by the translation machinery and consequently promoting or inhibiting translation from the IRES. Several different ITAFs regulate p53α- and Δ40p53-related IRESs, which are responsible for the relative expression of these two isoforms [173]. Therefore, identification and targeting of ITAFs that regulate p53 isoform expression could be a promising approach to modulate p53 isoform activities and improve anticancer treatment. Besides ITAFs, indirect modulation of p53 isoform expression by specific factors could also provide a therapeutic strategy. Sun and colleagues showed that netrin-1, an anti-apoptotic protein often overexpressed in aggressive cancers, can be transcriptionally regulated by Δ40p53, which binds to and activates its promoter [80]. Inhibition of netrin-1 in the presence of Δ40p53 induces cell death in tumor and primary cells and inhibits tumor growth in vivo. A positive correlation between netrin-1 and Δ40p53 gene expression was observed in human melanoma and CRC biopsies, and it is known that netrin-1 and its receptors are involved in the progression of these two types of cancer. Finally, netrin-1 interference presents a promising therapeutic strategy in tumors with upregulated Δ40p53 [80]. Similarly, Δ133p53 activates the JAK-STAT and RhoA-ROCK pathways, which promote migration and invasiveness of CRC cells. Thus, in patients with high ∆133p53 expression, molecules targeting these pathways may be used as therapy [40].

The p53 protein is mutated or functionally inactivated in most human cancers. Although *TP53* mutations affect p53 isoforms in the vast majority of *TP53*-mutant cancers, their impact on the biological activities of individual isoforms remains poorly understood. Importantly, several clinical studies have demonstrated that combining *TP53* mutation status with p53 isoform expression profiles improves the prognostic assessment of cancer patients [79,92,94,101,102,174,175]. Notably, although *TP53* mutations in cancer predominantly cluster within the DNA-binding domain, codons 100–132, which are not coding for the Δ133p53α isoform, show approximately twofold-fewer mutations per codon compared with codons 133–300. This distribution suggests a selective advantage for mutations affecting regions shared by both full-length p53 and Δ133p53 isoforms, potentially disrupting the DNA-binding activity of both proteins simultaneously [176]. Furthermore, a recently described *TP53*β stop-lost variant was shown to alter p53 transcriptional activity, potentially promoting anti-apoptotic and pro-survival functions and thereby contributing to cancer predisposition [56]. To date, only a limited number of studies have investigated the role of mutant p53 isoforms in tumor biology. In this context, the recent study by Joruiz and colleagues is particularly noteworthy [160]. The authors demonstrated that the mutant Δ133p53α R273H isoform promotes proliferation, invasion, genomic instability, and inflammation while suppressing apoptosis and cell cycle arrest genes in glioblastoma cells, thereby identifying mutant Δ133p53α as an active contributor to glioblastoma carcinogenesis and response to treatment. Similarly, Δ133p53β isoform carrying *TP53* mutations may also contribute to mutant p53 GOF activities [26,176]. In addition, Candeias and colleagues have found that Δ160p53 isoform is frequently overexpressed in cancer cells harboring hotspot *TP53* mutations contributing to GOF phenotype. Furthermore, downregulation of endogenous Δ160p53 in R273H mutant A431 and HT29 cells results in abrogation of GOF, providing a potential mechanism for targeting mutant p53 activities [177]. Together, these findings emphasize that understanding the functional consequences of *TP53* mutations across distinct p53 isoforms represents an important yet still insufficiently explored area of p53 research.

In tumors retaining wild-type *TP53*, MDM2 upregulation is one of the mechanisms of p53 functional inactivation. as MDM2 promotes p53 protein degradation [178]. Interestingly, the p53β and p53γ isoforms are less susceptible to MDM2-mediated degradation, with nonsense-mediated decay (NMD) representing the main pathway for degradation of these two isoforms [179]. Strategies for p53 reactivation are currently under investigation. One of the recently recognized approaches for restoration of p53 tumor-suppressive function is NMD inhibition, which has been shown to upregulate p53β and p53γ in tumor cells harboring *TP53* mutations downstream of exon 9 or overexpressing MDM2, thereby restoring activation of the p53 pathway. NMD inhibition also promotes apoptosis, reduces cell viability, enhances tumor radiosensitivity, and suppresses tumor growth in xenograft tumor models [179]. In addition, Δ133p53α levels can be regulated by autophagic degradation. Both Δ133p53α and the chaperone-associated E3 ubiquitin ligase STUB1, which regulates autophagy, are downregulated during replicative senescence. Therefore, siRNA knockdown of STUB1 induces autophagic degradation of Δ133p53α and promotes senescence [180]. This study demonstrates that STUB1 acts as a regulator of both Δ133p53α degradation and senescence, and that p53 isoforms are associated with two key cellular processes involved in aging and cancer [181]. Collectively, these results indicate that regulating p53 isoform expression through degradation mechanisms offers a novel therapeutic strategy. Aggregation mechanisms also contribute to p53 isoform regulation. Mutant p53 can promote aggregation of WT p53, which leads to dominant-negative and GOF effects that increase cancer aggressiveness and progression [181,182]. Aggregation tendency of p53 protein is related to the absence of DBD that is frequently mutated in p53 or is partially missing as in Δ133p53 isoforms. Molecular dynamics studies proposed that Δ133p53β has a higher aggregation tendency than p53β, and that a peptide with the same amino-acid sequence as fragments from the truncated DBD region (p53 residues 107–129) can restore Δ133p53β to the WT p53 conformation, consequently restoring p53’s tumor suppressor activity [17]. If validated in vivo, such approaches could have therapeutic potential. In conclusion, strategies for reactivating p53 by using p53 isoforms as targets provide potential for cancer therapies.

Dysregulation of alternative mRNA splicing is another hallmark of cancer, making splicing factors promising treatment targets [183,184,185]. Phosphorylation of serine/arginine-rich (SR) splicing factors (SRSFs) has been shown to affect isoform production, so molecules influencing this process may have therapeutic potential [186,187]. SRSF7 is a positive regulator of the alternative splicing pathway, while SRSF1 and SRSF3 inhibit alternative splicing through competitive binding at the intron 9 site [188,189]. Cooperation between SRSF1 and p53 has been demonstrated in vitro in 18 cancer cell lines and 85 breast cancer specimens, indicating possible clinical significance [188]. SRSF3 binds to the alternatively spliced exon unique to p53β, preventing the inclusion of this alternative exon in the fully spliced p53 transcript [189]. Normal human cells capable of entering replicative senescence have lower levels of SRSF3 and higher levels of p53β. Moreover, SRSF3 overexpression has been observed in numerous tumors, supporting its role in inhibiting senescence and promoting tumorigenesis, perhaps in part through downregulation of p53β [190]. Caffeine is a small molecule that decreases expression of SRSF3 and p53α while increasing p53β expression, which enhances cellular senescence [189,191]. Besides SR proteins, inhibition of SR-protein-specific kinases, such as Cdc2-like kinases (Clks), may also have therapeutic value. The specific small-molecule Clks inhibitor TG003 induces the expression of p53β and p53γ at both mRNA and protein levels in MCF7 breast cancer cell line and promotes cell apoptosis [188]. Other Clks inhibitors, such as KH-CB19 and GPS167, also prevent phosphorylation of splicing factors, indicating possible clinical applications [192,193]. In addition, protein phosphatases, which impair canonical splicing via dephosphorylation of SRSFs, might affect the generation of p53 C-terminal isoforms [194]. Along with small-molecule inhibitors, splice-switching antisense oligonucleotides (SSOs) and the CRISPR-Cas strategies are promising therapeutic approaches developed to target splicing factors [183,185]. However, both are currently being investigated for their potential clinical use.

Precise determination of p53 isoform expression is the cornerstone for clinical translation which can potentially enable selective targeting of p53 isoforms in the future. Several scientific tools have been developed to investigate the expression of p53 isoforms, either on mRNA or protein level. On the mRNA level, RT-qPCR protocols can either include TaqMan chemistry to determine groups of isoforms or the very reliable method that is based on nested PCR [7,8,195]. Nested PCR consists of two reactions; the first amplifies regions specific for long or short isoforms and the second includes qPCR reaction with primer sets that are specific for each isoform. Recently, a multiplex long amplicon ddPCR method was established which enables precise end-to-end quantitation of the seven *TP53* transcripts, with amplicons ranging from 0.85 to 1.85 kb [196]. Although extremely efficient, neither of these methods are capable of distinguishing between Δ133p53 and Δ160p53 isoforms since both are encoded by the same *Δ133p53* mRNA transcript [197]. On the protein level, different monoclonal antibodies have been developed to detect endogenous p53 isoforms by immunofluorescence, immunohistochemistry and Western blotting. Each antibody is designed to bind specific epitopes and thus recognizes certain groups of p53 isoforms. In addition to the limitation of not being able to detect individual isoforms, determination of isoforms at the protein level can be hampered by the presence of post-translational modifications on the epitope that can interfere with antibody binding [7,8].

Importantly, mRNA and protein levels sometimes do not correlate; therefore, determination and quantification of isoforms on both levels should be taken into consideration when investigating the expression status in tumor samples or biological activities in cancer cell lines. This could especially be challenging in clinical settings due to small cohort sizes and limited tissue material, so additional efforts should be invested into developing fast, optimized and precise methodology to detect p53 isoforms in tumor tissue.

## 4. Conclusions

The *TP53* gene, recognized as a pivotal tumor suppressor, gives rise to multiple isoforms that exhibit overlapping but also distinct functions. These isoforms may act either in concert or independently of the canonical full-length p53 protein, and this complexity should be carefully considered when investigating the role of p53 in cancer development. Numerous studies have demonstrated their involvement in tumor development and therapeutic response. In particular, N-terminally truncated isoforms have attracted considerable attention as important modulators of the p53 function. Specifically, Δ40p53 and Δ133p53 isoforms are frequently upregulated in various cancers, although functional effects vary among tumors. Functional and biological significance connected to imbalanced expression pattern of p53 isoforms is still ill comprehended and needs elucidation. Despite challenges, ongoing efforts to fully resolve biological roles of p53 isoforms are important for a comprehensive understanding of tumor biology. In particular, targeting specific p53 isoforms with pro-oncogenic activities represents an important direction for future research and for the development of novel therapeutic strategies.

## Figures and Tables

**Table 1:** The expression and biological functions of p53 isoforms in different cancers.

Cancer	Isoforms	Description	Reference
Colorectal cancer	p53β	Changes expression during cancer progression, i.e., increased levels in premalignant lesions of colon adenomas compared to normal colon tissue and decreases expression from stage II to III.	[34,45]
		Depletion reduces invasion of HCT116 cells.	[40]
		Potential predictive biomarker in familial CRC.	[56]
	Δ40p53	Negatively regulates the expression of YY1 protein and inhibits proliferation of p53-null HCT116 cells.	[53]
		Inactivates autophagy and cell death in Δ40p53 expressing HCT116 cells.	[54]
	Δ133p53α	Reduced levels in premalignant lesions of colon adenomas compared to normal colon tissue.	[34,45]
	Δ133p53β	Inhibits camptothecin-induced apoptosis by binding to a tumor suppressor RhoB.	[39]
	Δ133p53	Changes expression during cancer progression, i.e., increases from stage I to II.	[34]
		Increased expression is associated with more advanced disease, shorter DFS and increased aggressiveness, higher metastatic potential in rectal cancer.	[39,40]
		Promotes expression of DNA-repair genes synergistically with p73.	[52]
		Ectopic expression increases levels of interleukin 6 and interleukin 8 as well as protein Bcl-2, initiates epithelial–amoeboid transition and promotes invasion via RhoA-ROCK pathway in HCT116 cells.	[26,40,55]
	p53β/Δ133p53	Expression pattern of high/low isoform levels is associated with senescence.	[34]
	Δ133p53/p53α	Higher ratio is associated with more advanced disease.	[34]
Melanoma	p53β	Expressed in melanoma cells and induced by cisplatin treatment.	[69]
		Reduced expression correlated with lower OS.	[37]
		Elevated cytoplasmic and nuclear staining correlate with advanced disease stage and reduced patient survival.	[82]
	Δ40p53	Expressed in melanoma cells.	[69]
		Interacts with FLp53 and promotes cell survival by activating netrin-1.	[80]
		Promotes apoptosis rather than cell cycle arrest, mediated by increased PIDD promoter occupancy and reduced expression of p21.	[75]
		Reduced expression is linked to less aggressive phenotypes, yet to shorter metastasis-free survival.	[82]
	Δ40p53β	Elevated expression in vemurafenib-resistant compared to vemurafenib-naïve melanoma cell lines.	[76]
	Δ133p53α	Elevated levels in metastatic melanoma compared to healthy tissue.	[37]
	Δ133p53β	Higher levels correlate with shorter OS of metastatic melanoma patients.	[37]
		Elevated levels associated with higher probability of recurrence and brain metastasis.	[79]
	Δ160p53α	Elevated levels in metastatic melanoma compared to healthy tissue.	[37]
	Δ160p53	Associates with chromatin to drive proliferation and migration.	[76]
Breast cancer	p53β	Higher expression associated with smaller tumor size and increased DFS.	[92]
		High cytoplasmic level is related to worse prognosis.	[93]
	p53γ	Lowest expression compared to other isoforms.	[92]
		Beneficial effect on the prognosis of breast cancer patients carrying *TP53* mutation.	[94]
		Higher expression positively correlates with lower tumor grade.	[92]
	Δ40p53	Increased expression in tumor compared to healthy tissues.	[92]
		High levels correlate with downregulation of differentiation-related genes, and upregulation of genes related to stem cell regulation in invasive ductal carcinoma. Overexpression increases expression of stem cell and EMT markers, such as *SOX2*, *OCT4*, *NANOG*, *ZEB1* and *CDH1* in MCF-7 cells. Higher levels correlate with increased mammosphere and colony formation abilities as well as downregulation of miR-145, miR-200a, and miR-200b.	[32]
	Δ40p53:p53α	Increased ratio correlated with impaired cell cycle regulation, decreased sensitivity to chemotherapeutics and inhibition of apoptosis-related genes. High ratio intensified tumor growth, Ki67 and *SOX2* expression, blood microvessel areas and resistance to doxorubicin in MCF-7 cells when injected in vivo.	[95]
	Δ133p53β	Promotes mammosphere formation, cancer cell stemness potential by positively regulating *SOX2*, *OCT3/4*, and *NANOG* expression and proportion of CD44^+^/CD24^−^ cells.	[31]
		Frequently expressed in tumors harboring mutant p53 compared to WT p53 tumors.	[26,96]
		Promotes cell invasion and increases the risk of cancer recurrence leading to decreased OS.	[26,96]
		Increased expression was found in brain metastases compared to primary breast tumors and is associated with reduced time for metastases to develop.	[79]
	Δ133p53	Expression is induced by chemotherapy (etoposide) treatment.	[31]
		Upregulates IFN-γ signaling which is associated with better patient outcome. Increased mRNA levels and IFN-γ signaling activity were specifically found in ER-positive (ER+) tumors carrying mutated *TP53*.	[97]
Ovarian cancer	Δ40p53	Increased expression correlated with increased RFS in patients with WT p53 and lower tumor grade of HGSOC.	[101]
		Increased expression in MOC compared to healthy tissues and is associated with better RFS.	[102]
	Δ133p53	Increased expression correlates with better RFS and OS in HGSOC patients carrying *TP53* mutation. Low levels in HGSOC are associated with resistance to platinum-based therapy.	[101]
		Expression varies in different cancer subtypes, e.g., lowest in ENOC compared to MOC and serous cancer.	[102]
		High levels in HGSOC correlate with improved OS.	[103]
Endometrial (uterine) carcinoma	p53γ	Higher expression correlates with shorter PFS.	[108]
	Δ40p53	Primarily detected in the cytoplasm in the form of amyloid aggregates that can modulate p53 cellular functions.	[109]
Prostate cancer	Δ40p53	Increased levels are associated with good patient prognosis.	[117]
	Δ133p53	Expression is stimulated by hypoxia and induces expression of *VEGFA* and *VEGFB* in 22Rv1 cell line.	[117]
	Δ133p53β	Elevated levels in tumors with high immune cell infiltration and increased proliferation as well as immunosuppressive characteristics. Associated with more aggressive form of cancer and shorter PFS. Regulates expression of *PD-L1*, *IL6ST*, *STAT6* and *CXCR6* in 22Rv1 cell line.	[117]
Lung cancer	∆133p53	Overexpressed in tumor compared to healthy tissue.	[42]
Head and neck squamous cell carcinoma	p53β	Most identified isoform in both tumor and normal tissue.	[121]
Gastric cancer	p53β/Δ133p53	Expression pattern changes (decreases/increases) during cancer progression.	[41]
	p53β	Induced expression upon cisplatin treatment in WT p53 MKN45 cells, which is enhanced after rmhTNF, but remains unaffected in mutant p53 SGC-7901 cells.	[130,131]
	∆133p53	Reduced expression after rmhTNF treatment.	[132]
		Reduced expression after PDTC treatment alone or when combined with cisplatin in MKN45 cell line.	[133]
		Increased expression upon *Helicobacter pylori* infection of gastric epithelial cells (AGS and SNU-1). Regulates expression of p53-target genes and expression of NF-κB targets in *Helicobacter pylori-*infected cells. Regulated via AP-1 transcription factor.	[55]
	∆160p53	Increased expression upon *Helicobacter pylori* infection of gastric epithelial cells (AGS and SNU-1).	[55]
Hepatocellular carcinoma	Δ40p53α	Overexpression induces senescence and suppresses colony formation as well as proliferation.	[140]
Cholangiocarcinoma	Δ133p53/p53	Higher expression ratio correlates with poor OS.	[43]
	Δ133p53	Associated with resistance to 5-FU in KKU-M214R and KKU-M139R cell lines. Suppression restores sensitivity to 5-FU and promotes apoptosis.	[30]
Renal cancer	p53β	Overexpressed in tumors compared to normal tissue.	[150]
		Expression decreases with advanced tumor stage. Higher expression is associated with improved OS and RFS.	[152]
	Δ40p53	Elevated expression is associated with larger tumor size and renal capsular invasion. Upregulated in mutant p53 tumors.	[153]
	Δ40p53α and Δ40p53γ	Upregulated in advanced stage tumors.	[151]
	Δ133p53	Downregulated in early-stage tumors.	[151]
		Downregulated in WT p53 tumors compared to healthy tissue. Upregulated in mutant p53 tumors.	[153]
Glioblastoma	Δ40p53	Expressed in tumor compared to non-tumor cerebral cortex tissue. Increased expression in neural progenitor cells of the brain and neurospheres compared to adherent monolayer.	[158]
	∆133p53	Positively regulates cell migration without affecting proliferation of U87 cells.	[28]
		Reduces sensitivity to temozolomide and promotes survival under oxidative stress.	[159]
	Δ133p53α	Overexpression stimulates tubulogenesis in vitro while downregulation inhibits angiogenesis and tumor-growth in vivo.	[28]
		Promotes RAD51 expression and DNA repair. Combined with p53 mutation p.R273H promotes proliferation, expression of EMT markers and invasiveness in 2D and 3D models.	[160]
		Reduces cellular senescence.	[159,160]
	Δ133p53β	Elevated levels in tumors with WT p53 and high content of infiltrating immune cells. Expressed in malignant cells in hypoxic tumor area. Contributes to immunosuppressive signature, e.g., regulates expression of *CCL2* thus CD163 macrophage infiltration, and increases both *CSF1R* and *PDL1* levels.	[159]
Sarcoma	p53β	Overexpression reduces DNA synthesis and proliferation of SAOS-2 cells.	[161]
	Δ133p53	Expression is induced upon doxorubicin treatment in U2OS cells.	[162]
		Promotes cell migration and invasion in vitro, and formation of lung metastasis in vivo.	[27]
		Higher levels in EWS. Positively regulates HGF expression and secretion.	[163]
Leukemia	p53β and p53γ	Correlate with long-term survival of AML patients and good prognosis after chemotherapy.	[168]
		Higher levels correlate with less differentiated stage AML.	
		Lower levels correlate with sensitivity of primary AML cells to VPA.	[169]
		Downregulation upon VPA treatment in MOLM-13 and MV4-11cells.	
	FLp53: p53β/γ	Higher ratio correlates with worse clinical outcome of CLL patients.	[170]
	p53β and Δ133p53	Increased levels in primary and relapsed BCP-ALL.	[171]
	Δ40p53	Increased levels in relapsed BCP-ALL.	[171]

Abbreviations: WT (wild-type), DFS (disease-free survival), PFS (progression-free survival), RFS (recurrence-free survival), OS (overall survival), IFN-γ (interferon-gamma), rmhTNF (recombinant mutated human tumor necrosis factor), PDTC (pyrrolidine dithiocarbamate), -FU (5-fluorouracil), EWS (Ewing sarcoma), HGF (Hepatocyte growth factor), VPA (valproic acid), AML (acute myeloid leukemia), BCP-ALL (B-cell precursor acute lymphoblastic leukemia), CLL (chronic lymphocytic leukemia).

## Data Availability

No new data were created or analyzed in this study. Data sharing is not applicable to this article.
